# Effect of α-cypermethrin and pirimiphos-methyl on wing morphology of *Tribolium castaneum* (Herbst) and *T. confusum* Jacquelin du Val: a comparative study

**DOI:** 10.1007/s11356-023-30783-3

**Published:** 2023-11-30

**Authors:** Vladimir Žikić, Maja Lazarević, Saša S. Stanković, Marijana Ilić Milošević, Nickolas G. Kavallieratos, Anna Skourti, Maria C. Boukouvala

**Affiliations:** 1 Faculty of Sciences and Mathematics, Department of Biology and Ecology, University of Niš, Višegradska 33, 18000 Niš, Serbia; 2 Laboratory of Agricultural Zoology and Entomology, Department of Crop Science, Agricultural University of Athens, 75 Iera Odos Str, 11855 Athens, Attica Greece

**Keywords:** Stored-product pests, Insecticides, Geometric morphometrics, Elytra, Hindwings, Shape changes

## Abstract

*Tribolium castaneum* (Herbst) and *Tribolium confusum* Jacquelin du Val (Coleoptera: Tenebrionidae) are widespread and serious pests of stored products. Various insecticides are applied aiming to effectively manage both species. Here, two insecticides are tested, the pyrethroid α-cypermethrin and the organophosphate pirimiphos-methyl, hypothesizing that they can lead to morphological changes in the certain body parts of the adult offspring of treated *T. castaneum* and *T. confusum* parental female adults. For this purpose, the geometric morphometric method to the elytra and hindwings was applied. Both males and females were included in the analysis. The results showed that adult individuals of *T. confusum* showed higher tolerance to both insecticides compared to *T. castaneum* adults. This finding is reflected in analyses of both pairs of wings in *T. confusum* where changes in shape were negligible. The hindwings of *T. castaneum* experienced deformations to both insecticides. More significant changes in wing shape were observed in the α-cypermethrin treatment compared to pirimiphos-methyl. In the case of *T. castaneum*, even the shortest exposure to insecticides (5 min) is enough to provoke shape changes in the hindwings. Deformities in offspring, caused after treatment of their parents with insecticides, could moderate the frequency of insecticidal applications in storages.

## Introduction

Insect wings are part of the exoskeleton with many functions, such as flying, courtship, communication, and avoiding natural enemies (Wootton 2009). The elytron is a transformed, hardened forewing of coleopterans, whose main role is the protection of the abdomen and hindwings (Saito et al. 2017; Song et al. 2021). Although coleopterans have well-developed hindwings, many species of the families Curculionidae, Dermestidae, and Carabidae are poor flyers (Peacock 1993; Plarre 2010; Imura et al. 2018), while several species of the families Bostrychidae, Cerambycidae, and Scarabaeidae are strong flyers (Dissanayaka et al. 2020; Floate 2021; El-Shafie et al. 2022). In the case of the closely related species *Tribolium castaneum* (Herbst) and *Tribolium confusum* Jacquelin du Val (Coleoptera: Tenebrionidae), only *T. castaneum* is able to fly (Ridley et al. 2011; Gurdasani et al. 2019).

Both species are serious secondary pests of stored products worldwide, causing extensive damage (Hill 2003; Rees 2004; Robinson 2005; Kumar 2017; Sedighi et al. 2019; Deb and Kumar 2021). Both species occur in various processing facilities, such as bakeries, mills, pet shops, and retail stores (Hagstrum and Subramanyam 2009), and cause damage to packaged food products (Mullen et al. 2012; Stejskal et al. 2017; Scheff and Arthur 2018). *Tribolium castaneum* is a polyphagous pest of stored products infesting 246 commodities, whereas *T. confusum* infests 138 commodities (Hagstrum et al. 2013). Apart from the direct losses of stored commodities caused by the feeding activity of both species, quantitative and qualitative downgrade is observed from their excreta and body fragments (Nowaczyk et al. 2009; Aslam et al. 2019). Species of the genus *Tribolium* are known to secrete certain toxic quinones that contaminate flour and related products, posing significant risks to public health (Ladisch et al. 1967; Yezerki et al. 2004; Robinson 2005; Krinsky 2019). One other serious issue that points out the importance of both species is the difficulty of their control due to their tolerance or resistance to various insecticides (Zettler and Cuperus 1990; Zettler 1991; Bossou et al. 2015; Attia et al. 2020). Therefore, the successful management of both *Tribolium* species is imperative.

Pirimiphos-methyl is an organophosphate insecticide with a wide range of uses, especially for the protection of stored grains worldwide (Redlinger et al. 1988; Pražić Golić et al. 2017). This insecticide targets the enzyme acetylcholinesterase (AChE), causing its phosphorylation, which is responsible for the hydrolysis of acetylcholine in the synaptic cleft of the neural system (O’Brien 1967; Donarski et al. 1989; Eleršek and Filipić 2011; Khan 2021). Pirimiphos-methyl has shown high efficacy against several stored-product insects (Huang and Subramanyam 2005; Kljajić and Perić 2007; Kavallieratos et al. 2017, 2019; Boukouvala and Kavallieratos 2020). For instance, adults of *Tenebrio molitor* L. (Coleoptera: Tenebrionidae), were all dead on treated wheat and maize with pirimiphos-methyl 14 days post-exposure (Kavallieratos et al. 2019).

The pyrethroid α-cypermethrin is used against a wide range of agricultural and public health-importance insect pests (EPA 2020). It disrupts the insect’s nerve membrane, causing inactivation or delayed closing of voltage-sensitive sodium channels. As a result, the duration of the opened sodium channels increases beyond normal, from a few milliseconds to seconds (abnormal) (Clark and Brooks 1989; Vijverberg and van den Bercken 1990; Kašuba et al. 2022), allowing more sodium ions to cross and depolarize the nerve membrane, which finally leads to neurotoxicity (Mohammadi et al. 2019; Ravula and Yenugu 2021). The insecticide α-cypermethrin has been evaluated against several stored-product insects (Athanassiou et al. 2004; Kavallieratos et al. 2017; Boukouvala and Kavallieratos 2020; Amjad et al. 2022). For instance, the immediate mortality of *Prostephanus truncatus* (Horn) (Coleoptera: Bostrychidae) adults, exposed in treated polypropylene storage bags with the label dose of α-cypermethrin reached 100%, after 5 days (Kavallieratos et al. 2017). Exposure of *Trogoderma granarium* Everts (Coleoptera: Dermestidae) eggs to the surface of concrete sprayed with α-cypermethrin at label dose reduced their hatchability to 6.7% in comparison with control dishes (100%) (Boukouvala and Kavallieratos 2020). The authors also recorded complete mortality of newly emerged larvae after 6 and 4 days, in the dishes with or without food, respectively.

Previous research efforts have revealed that the exposure of insects to various toxic substances may have a direct impact on their development and morphology (Rodríguez Enríquez et al. 2010; Khan et al. 2016; Liu et al. 2018) or indirectly affect their offspring (Mondal and Parween 2000; Mohandass et al. 2006; Lazarević et al. 2019; Skourti et al. 2021a, b). For instance, newly hatched larvae of *Spodoptera frugiperda* (J.E. Smith) (Lepidoptera: Noctuidae), showed body deformations at all stages of the life cycle after being fed with corn treated with ZnO nanoparticle solution at different concentrations (Pittarate et al. 2021). Bernardes et al. (2018) reported malformations in queens of *Partamona helleri* (Friese) (Hymenoptera: Apidae) when exposed orally to diets containing four doses of azadirachtin during their development. The number of individuals showing deformations increased with increasing dose, displaying deformities in the reproductive system, mandibles, antennae, wings, and legs.

Regarding the indirect effects of insecticides on the next generation of exposed insects, a recent study has documented that pirimiphos-methyl causes deformities on the fore and hindwings of the offspring when adult females of *T. granarium* treated with this organophosphorus insecticide (Lazarević et al. 2019). The method of geometric morphometrics was used to determine changes in the wings invisible to the naked eye that occurred as a result of treating individuals with pirimiphos-methyl. This method is increasingly used to detect minute changes in the morphology of various anatomical structures, especially insect wings. As two-dimensional structures, wings are particularly suitable for the application of geometric morphometrics (Žikić et al. 2009; Cvetković et al. 2020; Champakaew et al. 2021; Farsi et al. 2022; Gu et al. 2022). However, the elytra of coleopterans are three-dimensional, and very difficult to orient in the same way for comparison, so special caution is required. Former studies have revealed that elytra can also be used to detect differences between or among coleopteran groups, e.g., Lucanidae subfamilies, *Colophon* spp., populations of *Brontispa longissima* (Gestro) (Coleoptera: Chrysomelidae), and *Ceroglossus chilensis* Eschscholtz (Coleoptera: Carabidae) (Acevedo 2015; Eldred et al. 2016; Juache et al. 2018; Zhang et al. 2019).

After an extensive survey of the global literature, several studies have investigated various effects of α-cypermethrin and pirimiphos-methyl against both species (Athanassiou et al. 2004; Velki et al. 2014; Papanikolaou et al. 2021; Skourti et al. 2021a). Although there are reports of direct abnormalities in morphogenesis caused by pirimiphos-methyl in both species (Khan 1981; Mondal 1984; Rahman 1992; Kamaruzzaman et al. 2006), no data were found for α-cypermethrin, nor for the indirect effect of both insecticides on *T. castaneum* and *T. confusum* offspring. Despite the fact that insecticides affect parental individuals, it is also important to measure their effects on offspring (Boukouvala and Kavallieratos 2021). This is because stored-product insects exhibit elevated reproduction capacity, an issue that highly accelerates the degradation of commodities through their progeny (Hill 2003). Thus, the objective of this study was to use geometric morphometrics to detect morphological changes in the wings of the two selected *Tribolium* species. It is understood here that the morphology of adults of both sexes of the F_1_ generation reflects the effects of exposure of parental females at different intervals to α-cypermethrin and pirimiphos-methyl. Consequently, the forewings (elytra) and hindwings of male and female offspring of *T. castaneum* and *T. confusum* were analyzed.

## Materials and methods

### Rearing insects and commodities

The insect colonies used in the experimentation have been maintained at the Laboratory of Agricultural Zoology and Entomology, Agricultural University of Athens, since 2003. The initial populations of *T. castaneum* and *T. confusum* had been found in storage facilities in Greece. Both species were reared in a mixture of wheat flour and brewer’s yeast (5%), at 30 °C and 65% relative humidity (RH), in continuous darkness.

### Insecticides and bioassays

The following commercially available insecticides were used in the bioassays: Actellic EC, which is a microencapsulated formulation containing 50% pirimiphos-methyl as an active ingredient (a.i.) (provided by Syngenta, Anthousa, Greece), and Power SC, which is a suspension concentrate with 62.4 g/L α-cypermethrin (a.i.) (provided by Hybrid Hellas, Metamorphossis, Greece). Both insecticide formulations were tested at the label doses recommended for surface treatments, i.e., 0.05 mg a.i./cm^2^ for pirimiphos-methyl and 0.1 mg a.i./cm^2^ for α-cypermethrin. One day before the beginning of the tests, the bottom of 24 Petri dishes, with a surface area of 50.27 cm^2^, was covered with cement CEM I 52.5 N (Durostick, Aspropyrgos, Greece) creating a layer to be treated with the selected insecticides. Polytetrafluoroethylene (60 wt% dispersion in water) (Sigma-Aldrich Chemie GmbH, Taufkirchen, Germany) was then used to coat the upper inner wall of each dish to prevent insects’ escape. All dishes were sprayed with 1 ml of an aqueous solution, containing the concentration of α-cypermethrin (8 dishes) or pirimiphos-methyl (14 dishes) corresponding to the label dose of each formulation. This procedure was carried out with different AG-4 airbrushes (Mecafer S.A., Valence, France) for each a.i., creating a fine mist. Two additional dishes previously sprayed with distilled water with an AG-4 airbrush were served as controls.

Female adults of *T. castaneum* or *T. confusum* were left to mate for 3 days. Females of both species were > 5 days old, since they can lay fertile eggs after ~ 5 and 4 days after eclosion, respectively (Dawson 1964). Thereafter, 30 female adults of *T. castaneum* or *T. confusum* were transferred to each dish and exposed for 0 (control), 0.5, 3, 5, 8, 16, 24, and 36 h, at 30 °C and 65% RH, in total darkness. Preliminary tests showed that both species treated with α-cypermethrin die at exposure intervals longer than 20 min. Thus, in this case, 30 female adults of *T. castaneum* or *T. confusum* were placed in each dish for 0 (control), 5, 10, 15, and 20 min, at the above conditions. Subsequently, after the termination of each exposure interval, females of each treatment were transferred separately into glass vials with 12.5 cm height and 7.5 cm diameter, containing 30 g white hard wheat flour, and kept at the same conditions. Parental individuals remained in the vials for a period of 2 weeks, which is a sufficient period to obtain a satisfactory number of progeny (Hill 2003). After the emergence of adults, the determination of the sex of both species was performed according to Halstead (1963). Then, all sexed individuals of each treatment-exposure group were placed separately in plastic vials with 96% ethyl alcohol for preservation, until geometric morphometrics analysis.

### Wing dissection and taking photos

Twelve specimens were randomly selected from each treatment (Table 1) and transferred to distilled water for rehydration to facilitate the detachment of elytra and hindwings. The elytra were then placed on microscope slides. Since the hindwings of the coleopterans are folded, they must be pressed with coverslips to be correctly positioned for proper photography when placed on microscope slides. The wings prepared in this manner were photographed for geometric morphometric analyses. We used a Leica 2500 microscope with a Leica DFC490 camera (Leica Microsystems, Wetzlar, Germany) at 5 × magnification for elytra, and 10 × for hindwings. After the photographs were taken, the wings were returned to vials filled with 96% ethyl alcohol and deposited at the Faculty of Science and Mathematics, University of Niš.

**Table 1:** Elytra and hindwings of two *Tribolium* species analyzed by using the geometric morphometrics method

		*T. castaneum*				*T. confusum*			
Insecticide	Exposure	Groups				Groups			
		Elytra		Hindwings		Elytra		Hindwings	
α-Cypermethrin	Control	12 ♀	12 ♂	12 ♀	12 ♂	12 ♀	12 ♂	12 ♀	12 ♂
	5 min	12 ♀	12 ♂	12 ♀	12 ♂	12 ♀	12 ♂	12 ♀	12 ♂
	10 min	12 ♀	12 ♂	12 ♀	12 ♂	12 ♀	12 ♂	12 ♀	12 ♂
	15 min	12 ♀	12 ♂	12 ♀	12 ♂	12 ♀	12 ♂	12 ♀	12 ♂
	20 min	12 ♀	12 ♂	12 ♀	12 ♂	12 ♀	12 ♂	12 ♀	12 ♂
Pirimiphos-methyl	Control	12 ♀	12 ♂	12 ♀	12 ♂	12 ♀	12 ♂	12 ♀	12 ♂
	30 min	12 ♀	12 ♂	12 ♀	12 ♂	12 ♀	12 ♂	12 ♀	12 ♂
	3 h	12 ♀	12 ♂	12 ♀	12 ♂	12 ♀	12 ♂	12 ♀	12 ♂
	5 h	12 ♀	12 ♂	12 ♀	12 ♂	12 ♀	12 ♂	12 ♀	12 ♂
	8 h	12 ♀	12 ♂	12 ♀	12 ♂	12 ♀	12 ♂	12 ♀	12 ♂
	16 h	12 ♀	12 ♂	12 ♀	12 ♂	12 ♀	12 ♂	12 ♀	12 ♂
	24 h	12 ♀	12 ♂	12 ♀	12 ♂	12 ♀	12 ♂	12 ♀	12 ♂
	36 h	0 ♀	0 ♂	0 ♀	0 ♂	12 ♀	12 ♂	12 ♀	12 ♂

### Sample structure

We conducted all analyses involving both sexes of two *Tribolium* species, *T. castaneum* and *T. confusum*. The effects of two pesticides were tested on both the elytra and hindwings, resulting in four analyses: (1) elytra of specimens exposed to α-cypermethrin, (2) elytra of specimens exposed to pirimiphos-methyl, (3) hindwings of specimens exposed to α-cypermethrin, and (4) hindwings of specimens exposed to pirimiphos-methyl. The 36-h exposure time with pirimiphos-methyl was analyzed only for *T. confusum*, as no parental *T. castaneum* females produced offspring after their contact with this insecticide for 36 h.

### Analysis of wing shape changes

**Fig. 1:**
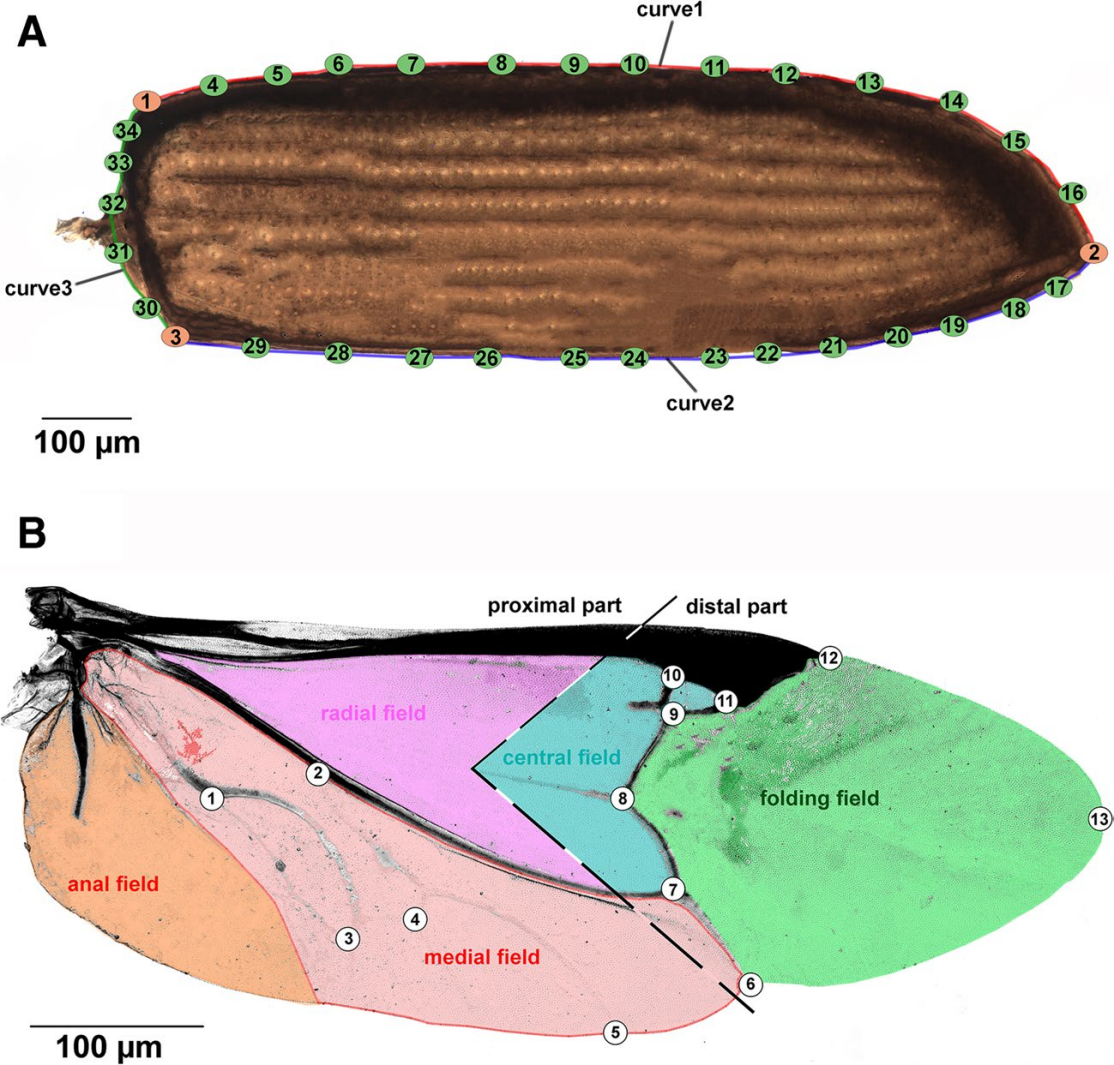
*Tribolium castaneum*, female, **A** elytron and **B** hindwing. The red circles on elytron are landmarks, whereas the green circles show the semi-landmarks which slide along 3 previously defined curves. The color represents the main hindwing fields. The dashed lines divided wings into proximal and distal parts. Wing vein abbreviations follow Forbes (1922) A, anal; Cu, cubital C, costal; M, medial; R, radial; RS, radial sector

Methods of geometric morphometrics were used to analyze the changes in the shape of both the elytra and hindwings (Zelditch et al. 2012). The shape of the elytra was determined by three fixed landmarks (LMs) that were used for the definition of 3 curves along which a series of equally spaced semilandmarks (S-LMs) slide along tangent vectors (Fig. 1A). The positioning of 34 specific landmarks (3 LMs and 31 S-LMs) on elytra was performed using StereoMorph 1.6.7 software (Olsen and Westneat 2015). To define the shape of the hindwing, we selected 13 true LMs. The proximal part of the wing is defined by LM6-13, the distal part of the wing by LM1-5, while the anal field of the wing is characterized by LM1-5. The folding field of the wing is illustrated by LM6-9 and LM11-13, and the radial sector field is specified by LM9-11 (Fig. 1B). The digitization of the hindwings was performed using tpsDig2 software (Rohlf 2018).

### Statistical analyses

To eliminate variations due to different positioning, scaling, and rotation, we applied generalized Procrustes analysis (GPA) for both analyzed structures, elytra and hindwings (Rohlf and Slice 1990; Dryden and Mardia 2016). By applying the GPA method, we obtain information about the wing shape in the form of Procrustes coordinates. To visualize the changes in wing shape, we applied principal component analysis (PCA).

The GPA and PCA methods mentioned above were performed using Geomorph version 4.0.5. (Adams et al. 2023). The results of the PCA were used to create scatter plots in ggplot2 version 3.4.3 software (Wickham 2016). With the Geomorph software, we conducted a multivariate analysis of variance (MANOVA) to test for significant differences in the shape of the analysed structures between species, between sexes, among treatments (i.e., categorical explanatory variables), and their interactions: between sexes within each species (whether wing shape was differently affected in males and females within each species) (species × sex), among treatments within each species (whether wing shape was differently affected by different treatments within each species) (species × treatment), among treatments within each sex (whether wing shape was differently affected by different treatments within each sex) (sex × treatment), and among treatments within each sex within each species (whether wing shape was differently affected by different treatments within each sex within each species) (species × sex × treatment).

## Results

### Analysis of elytra

**Fig. 2:**
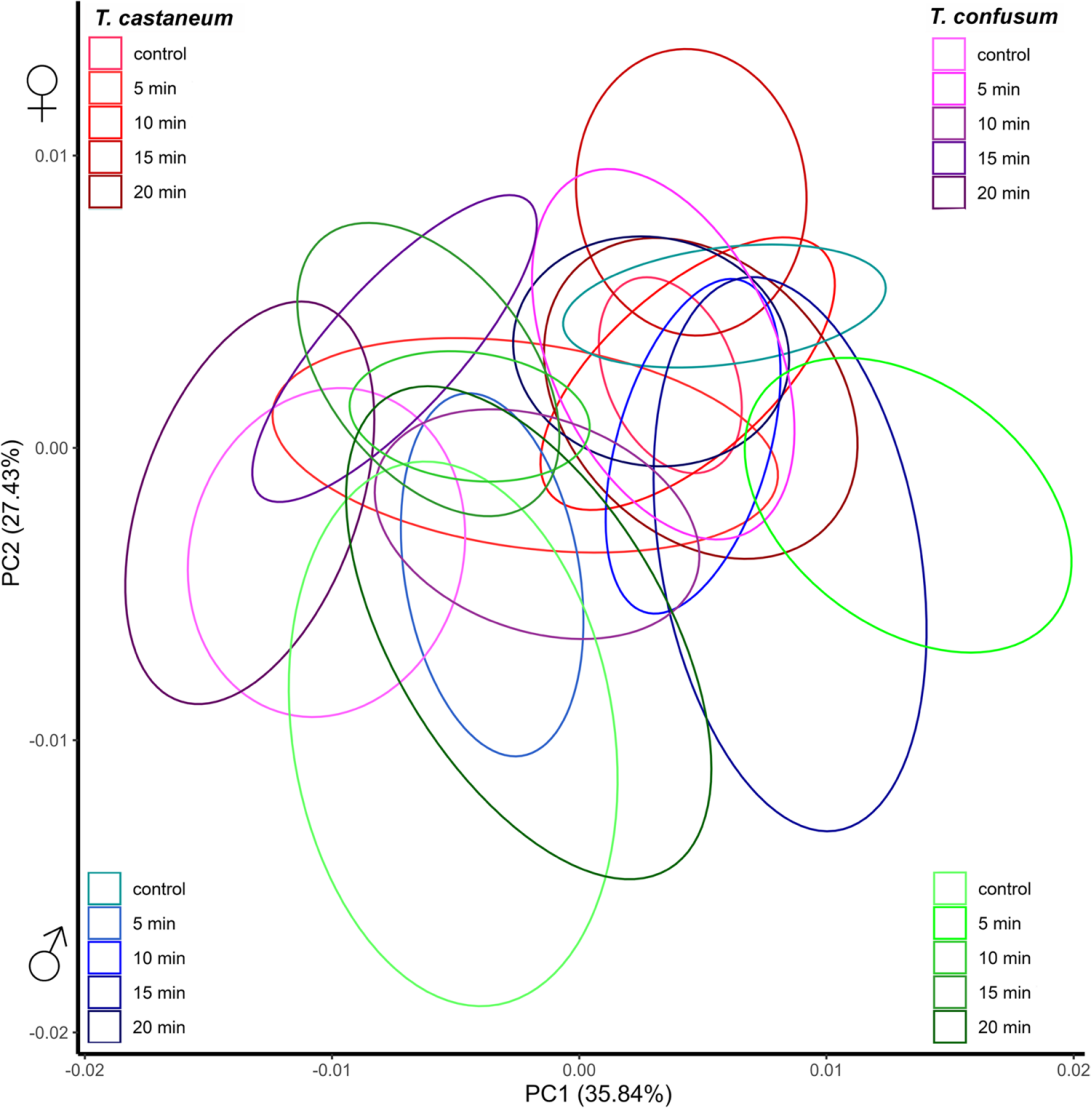
Comparative analysis of morphological changes on the elytra of *T. castaneum* females (red) and males (blue) and *T. confusum* females (purple) and males (green) under the effect of α-cypermethrin, displayed in a PCA morphospace. Ellipses represent a 90% confidence interval for groups’ means

The effects of α-cypermethrin on the elytra shape showed significant differences for species, sex, treatment, species × treatment, and species × sex × treatment (Table 2 (A)). Despite statistically significant results for the shape of the elytra, differences in the morphology of the elytra between the control and treatment groups were not noticeable. Results are displayed in the morphospace defined by the first two PC axes. Both tests exhibit high support for elytra, α-cypermethrin (PC1 + PC2 = 63.27%), and pirimiphos-methyl (PC1 + PC2 = 61.28%). This is observed for both species of *Tribolium* for both sexes (Fig. 2). Since the differences in the elytra shape between control groups and all the treatments are negligible, grids of deformations are not shown.

**Table 2:** MANOVA on shape changes caused by categorical explanatory variables for (A) elytra, under exposure to α-cypermethrin; (B) elytra, under exposure to pirimiphos-methyl; (C) hindwings, under exposure to α-cypermethrin; and (D) hindwings, under exposure to pirimiphos-methyl

Effect	DF	SS	MS	*Rsq*	*F*	*Z*	*P*
(A) Elytra: α-cypermethrin							
Species	1	0.002767	0.00276669	0.03788	10.5606	4.447	< 0.01
Sex	1	0.000993	0.00099305	0.0136	3.79505	2.5621	< 0.01
Treatment	4	0.0019	0.00047505	0.02602	1.8133	1.9931	0.02
Species × sex	1	0.000547	0.00054664	0.00748	2.0866	1.4867	0.07
Species × treatment	4	0.005958	0.00148953	0.08158	5.6856	6.08	< 0.01
Sex × treatment	4	0.001125	0.00028121	0.0154	1.0734	0.3817	0.35
Species × sex × treatment	4	0.002112	0.00052806	0.02892	2.0157	2.3192	0.01
Residuals	220	0.057636	0.00026198	0.78913			
Total	239	0.073038					
(B) Elytra: pirimiphos-methyl							
Species	1	0.0002182	0.0002182	0.01073	5.0473	2.3548	0.01
Sex	1	0.0000008	0.00000083	0.00004	0.0192	-2.0602	1.00
Treatment	7	0.0018419	0.00026313	0.09058	6.0866	6.4613	< 0.01
Species × sex	1	0.0000632	0.00006325	0.00311	1.4631	0.7464	0.24
Species × treatment	6	0.003137	0.00052283	0.15427	12.0941	9.2801	< 0.01
Sex × treatment	7	0.00027	0.00003857	0.01328	0.8922	-0.1623	0.57
Species × sex × treatment	6	0.0005801	0.00009668	0.02853	2.2364	2.337	0.01
Residuals	329	0.0142227	0.00004323	0.69946			
Total	358	0.0203339					
(C) Hindwings: α-cypermethrin							
Species	1	0.30995	0.309954	0.51147	384.0637	4.9214	< 0.01
Sex	1	0.007	0.007003	0.01156	8.6774	3.9612	< 0.01
Treatment	4	0.05345	0.013362	0.0882	16.5565	8.5096	< 0.01
Species × sex	1	0.00112	0.001116	0.00184	1.383	0.9484	0.17
Species × treatment	4	0.04771	0.011927	0.07872	14.7781	12.6262	< 0.01
Sex × treatment	4	0.00263	0.000657	0.00433	0.8136	-0.8565	0.81
Species × sex × treatment	4	0.00661	0.001651	0.0109	2.0462	3.4639	< 0.01
Residuals	220	0.17755	0.000807	0.29298			
Total	239	0.60601					
(D) Hindwings: pirimiphos-methyl							
Species	1	0.44253	0.44253	0.52574	542.3727	5.4313	< 0.01
Sex	1	0.00654	0.00654	0.00777	8.0203	4.5231	< 0.01
Treatment	7	0.05728	0.00818	0.06805	10.0288	11.882	< 0.01
Species × sex	1	0.00184	0.00184	0.00219	2.2599	2.1166	0.02
Species × treatment	6	0.05505	0.00917	0.0654	11.2442	13.4077	< 0.01
Sex × treatment	7	0.00532	0.00076	0.00632	0.9316	-0.3405	0.63
Species × sex × treatment	6	0.00635	0.00106	0.00755	1.2981	1.5523	0.06
Residuals	327	0.2668	0.00082	0.31697			
Total	356	0.84172					

*SS* sums of squares, *MS* mean squares, *Rsq R*^2^ (proportion of the variation in the wing shape that is related to each categorical variable), *Z* effect size

**Fig. 3:**
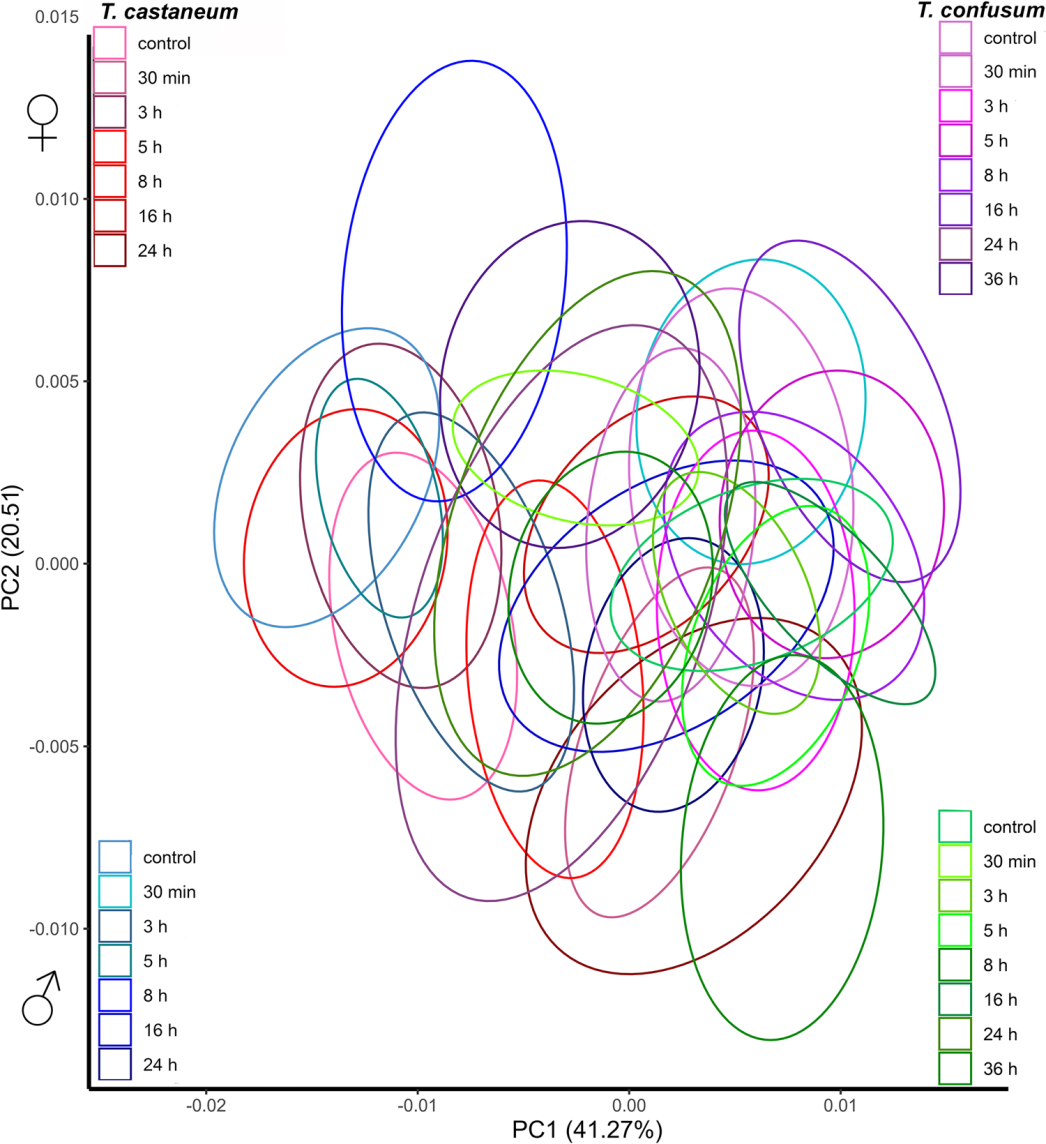
Comparative analysis of morphological changes on the elytra of *T. castaneum* females (red) and males (blue) and *T. confusum* females (purple) and males (green) under the effect of pirimiphos-methyl, displayed in a PCA morphospace. Ellipses represent a 90% confidence interval for groups’ means

Pirimiphos-methyl resulted in statistically significant variations in elytra shape for both *Tribolium* species, treatment, species × treatment, and species × sex × treatment (Table 2 (B)). Analogous to the effects of α-cypermethrin, elytra shape (for both species; males and females) discrimination in the morphospace defined by PC1 + PC2 axes was not recorded when treatments included pirimiphos-methyl (Fig. 3). As in the previous analysis, due to undetectable differences between the control and treatment groups, deformation grids are not shown.

### Analysis of hindwings

**Fig. 4:**
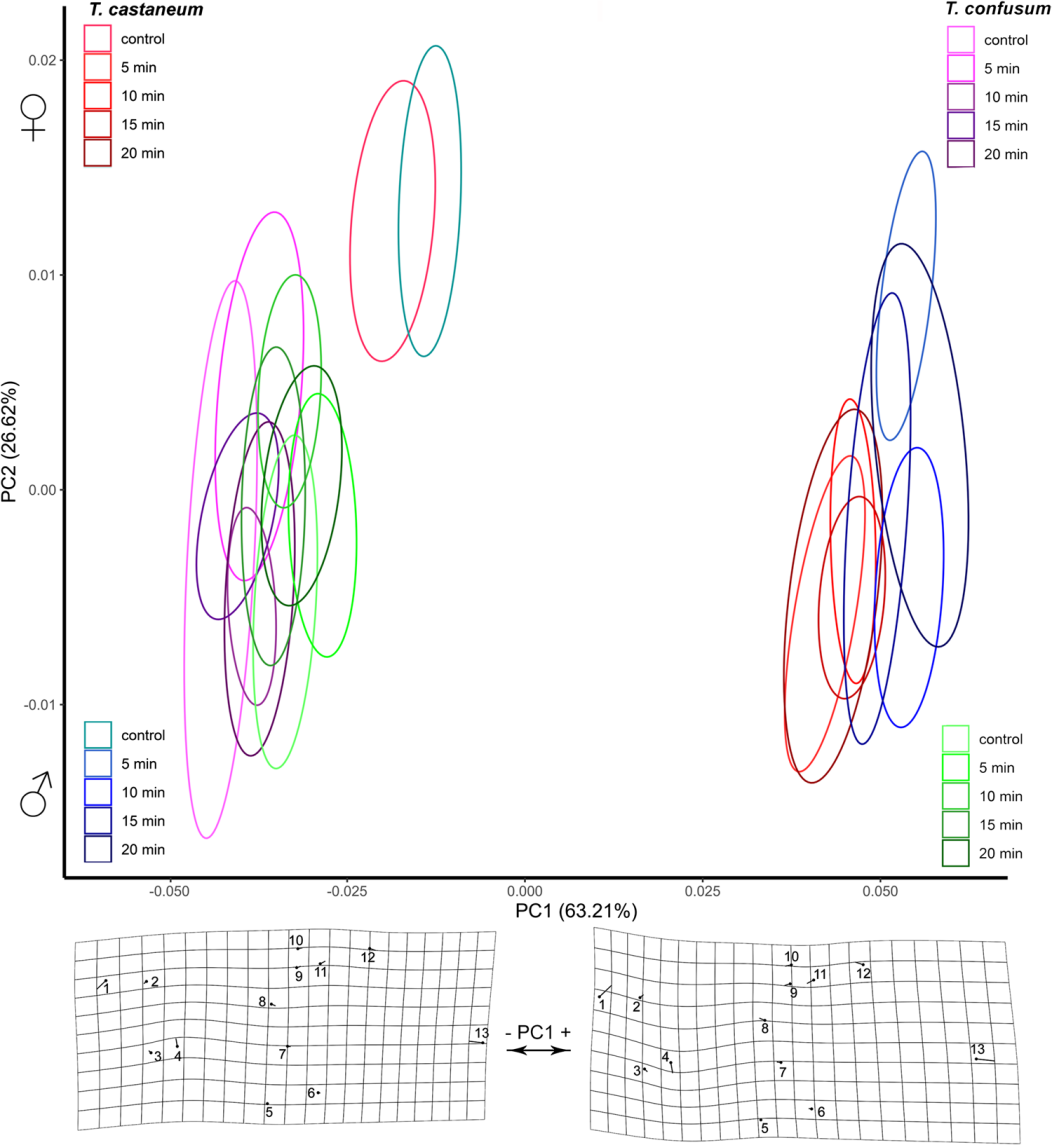
Comparative analysis of morphological changes of *T. castaneum* females (red) and males (blue) and *T. confusum* females (purple) and males (green) on the hindwings under the effect of α-cypermethrin, displayed in a PCA morphospace. Ellipses represent a 90% confidence interval for groups’ means. Deformation grids visualize the changes on the hindwings. The vectors show the magnitude of the change

**Fig. 5:**
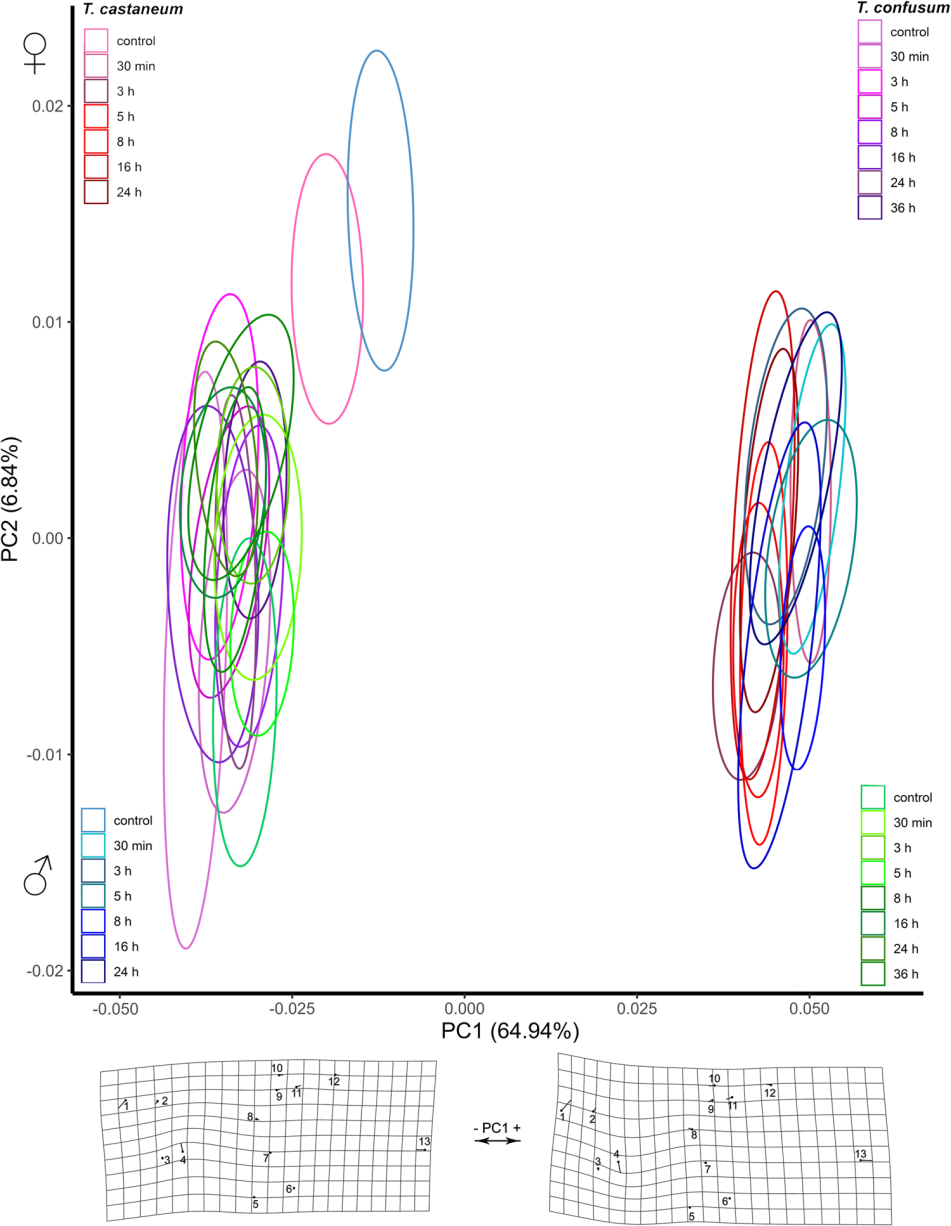
Comparative analysis of morphological changes on the hindwings of *T. castaneum* females (red) and males (blue) and *T. confusum* females (purple) and males (green) under the effect of pirimiphos-methyl, displayed in a PCA morphospace. Ellipses represent a 90% confidence interval for groups’ means. Deformation grids visualise the changes on the wings. The vectors show the magnitude of the change

After exposure of *Tribolium* parental females to α-cypermethrin, the variation in wing shape of their progeny was statistically significant. We observed highly significant results for species, sex, treatment, species × treatment, and species × sex × treatment (Table 2 (C)). In the morphospace defined by the first two PC axes (PC1 + PC2), there is a clear segregation of *T. castaneum* and *T. confusum* along the first PC axis, which in discrimination exceeds 63% (Fig. 4). Moreover, within *T. castaneum*, in both females and males, an indisputable separation of both male and female control groups from the pesticide-exposed groups was observed. This was noted in both treatments α-cypermethrin and pirimiphos-methyl (Figs. 4 and 5). For *T. castaneum*, both control groups, males and females are positioned far from the treatments. Control groups are close to *T. confusum* distributed along the negative part of PC1 (Fig. 4). As illustrated in Fig. 4, males are more affected than females. There is no effect of α-cypermethrin to hindwing shape in *T. confusum*, neither to females nor to males given that control groups are clustered together with treatments. For *T. castaneum* individuals, this is mainly indicated by LM8 and LM13 (Fig. 4). The radial field is shortened both in length and width (LM2, 8–10). The opposite changes are observed in *T. confusum* whose wings have decreased in both length and width, primarily by moving LM4 and LM13.

When the parental females of both *Tribolium* species were subjected to pirimiphos-methyl, significant results were obtained in the wing shape, similar to the outcome observed for α-cypermethrin (Table 2 (D)). Comparing these results with the results for the α-cypermethrin exposure, the response of individuals to the effects of pirimiphos-methyl is still high. Both control groups, males and females, are positioned closer to the treatments of *T. confusum*. The visualization of wing changes of *T. castaneum* and *T. confusum* is presented by the deformation of the transformation grid (Fig. 5). Comparing the control groups of *T. castaneum* with the insecticide exposure groups, it is noticeable that pirimiphos-methyl has affected mainly the folding and radial fields of the hindwing. The changes can be traced in Fig. 5.

Regarding the deformation grid, in the proximal part of the wing, LM1 and LM4 stand out the most in terms of intensity of affection. Certainly, the LM13 has a big share in the change of the shape of the wing. These hindwing changes were observed in both females and males. The changes are reflected in the lengthening of the folding field, i.e., the shortening of the radial hindwing field. The vectors show the magnitude of the change. The longer the vectors are, the more the grid deforms, which indicates greater changes in that part of the wing. The stable part of the wing consists of LM5-7, which describes the distal part of the anal field.

## Discussion

In the current study, wing morphological abnormalities of the F_1_ generation of *T. castaneum* and *T. confusum* were investigated after exposure of the parental females to α-cypermethrin and pirimiphos-methyl. Our results indicate that both insecticides act differently on changes in the shape of the forewings and hindwings of the selected *Tribolium* species. Comparing the data of these two insecticides, *T. castaneum* was more sensitive to their application in contrast to *T. confusum*. This is reflected in the fact that although *T. castaneum* females survived after 36 h of exposure to treated concrete with pirimiphos-methyl, they did not produce offspring. In a previous study, *T. castaneum* showed a higher susceptibility rate when exposed to deltamethrin powder applied to different surfaces (i.e., wood, concrete, and tile) than *T. confusum* (Arthur 1997). Similar results were observed by Scheff and Arthur (2018) for *T. castaneum* and *T. confusum* when exposed for long periods in deltamethrin-incorporated packaging. *Tribolium castaneum* did not survive longer than 48 h, while the first *T. confusum* individual died after 168 h. The same trend has been observed for the insect growth regulator methoprene, where *T. confusum* was shown to be more tolerant than *T. castaneum* (Arthur 2008; Tucker et al. 2014). All the above findings confirm that *T. castaneum* exhibits higher susceptibility than *T. confusum* under different scenarios. Furthermore, considering the inability of *T. castaneum* females to produce offspring after their exposure to pirimiphos-methyl for 36 h, we hypothesize that this species exhibited higher delayed mortality than *T. confusum*, confirming the susceptibility of *T. castaneum* to this insecticide.

Several studies have revealed the direct impact of insecticides in the deformations of insects when previously exposed as immatures. For example, when late-instar of *T. confusum* larvae was exposed for 12 weeks to methoprene (experimental formulation 202–084)-treated concrete at 32 °C and 75% RH, most emerging adults were dead and grossly deformed in morphology (Arthur and Hoernemann 2004). Pittarate et al. (2021) reported that when the first-instar larvae of *S. frugiperda* were fed on ZnO-treated corn, the width and weight of the emerged pupae were significantly lower than control pupae. The authors also noted that the fertility and fecundity of females derived from treated larvae, as well as the hatchability of the produced eggs, were significantly reduced in comparison to control (i.e., 1.92–7.64% of eggs were hatched for treated females vs. 96.4% of eggs were hatched for control females), showing the influence of ZnO on the reproductive capacity of this species. Concerning the indirect effects of insecticides on F_1_ generation, there is limited knowledge. Recently, Skourti et al. (2021a) reported that the exposure intervals of 24 and 72 h of parental females of *T. castaneum* treated with pirimiphos-methyl were crucial for the reproduction capacity of their offspring. Concretely, a significant reduction in fecundity of the exposed females was recorded after the aforementioned periods (4.5 and 4.8 females/female, for 24 and 72 h, respectively) in comparison to the control (17.0 females/female). The same trend was observed for the mean survival time, the intrinsic rate of increase, the finite rate of increase, and the values of doubling time. The most important finding of the current study deals with the confirmation of the results of Skourti et al. (2021a), indicating that the short exposure of *T. castaneum* parental females to treated concrete with the label dose of pirimiphos-methyl does affect the demographic parameters of the next generation and the morphology of their wings. Whether the detected morphological deformations of *T. castaneum* and *T. confusum* to α-cypermethrin coexist with injurious effects on the fitness of progeny merits further experimentation.

Interestingly, the thicknesses of elytra differ between *T. castaneum* and *T. confusum,* an issue that is related to their flight ability (Zohry and El-Sayed 2019). The middle part and the lateral edge of *T. confusum* elytra were thinner (i.e., 22.70 and 53.75 μm, respectively) in comparison to *T. castaneum* elytra (i.e., 27.0 and 54.54 μm, respectively). Moreover, considerable variations were observed in the space of the haemolymph in the elytra of both species. The strong flyer *T. castaneum* exhibited larger haemolymph spaces than the non-flying species *T. confusum*. Specifically, the height of the inner and lateral edges and the middle part were higher in *T. castaneum* (i.e., 16, 30.0, and 9 μm, respectively) than in *T. confusum* (i.e., 12.5, 18.75 μm, 4.55 μm, respectively) (Zohry and El-Sayed 2019). Despite the differences, the current study suggests that no significant separation exists between the distribution of elytra between insecticidal treatments and controls in the morphospace, indicating that the elytra of *T. castaneum* and *T. confusum* do not reflect the influence on the change of their morphology. This was observed at all tested exposures of parental females to α-cypermethrin and pirimiphos-methyl. In contrast, the application of pirimiphos-methyl to parental females of *T. granarium* led to greater sensitivity of the hindwings and lesser sensitivity of the elytra of their progeny (Lazarević et al. 2019).

Although females of *T. castaneum* and *T. confusum* seem to be larger than males (Park 1934; Krause et al. 1962), size is not always a consistent morphological character between the two sexes (Park 1934). The finding here reflects this phenomenon given that differences in the morphology of elytra and hindwings were not noticeable between males and females. In contrast, Lazarević et al. (2019) reported that when parental *T. granarium* females were exposed to pirimiphos-methyl, their female progeny suffered more robust deformations in elytra and hindwings in comparison to male progeny. It should be noted that *T. granarium* female adults are clearly larger (average 2.81 mm) than males (average 1.99 mm) (FAO 2016). Therefore, between larger females and smaller males of the same species, females are prone to express obvious deformities as a consequence of maternal exposure to toxicants (Lazarević et al. 2019). More pieces of evidence are needed to reveal whether this feature is constant among stored-product pests under the pressure of contact insecticides.

Deformations on the hindwings were detected in *T. castaneum* even at the exposure of 5 min for α-cypermethrin and at 30 min for pirimiphos-methyl. In both insecticidal experiments with *T. castaneum*, it was observed that the greatest changes in the wing plate occurred in the anal field in the proximal part and the folding field in the distal part. Morphologically, hindwings are membranous structures that are folded multiple times under the elytra when the insect is resting (Frantsevich 2011; Sun et al. 2018). Consequently, hindwings are much more complex than elytra (Frantsevich et al. 2005; Haas 2006). This can lead to increased possibility of malformations that occur during insect embryology, indicating that the toxic agent affected the eggs of the exposed females (Lazarević et al. 2019). Indeed, this phenomenon has been reported in the offspring of rodents after the exposure of parental females to pyrethroid insecticides during gestation. A reduction in sperm count, testicular weight, and epididymal weight was observed in the F_1_ generation (Zhang et al. 2021).

*Tribolium confusum* exhibited tolerance to α-cypermethrin and pirimiphos-methyl regarding the effects detected on the hindwings. No conspicuous changes were observed in the hindwing shape of this species. In an earlier study, Kamaruzzaman et al. (2006) allowed newly emerged larvae of *T. castaneum* and *T. confusum* to feed on treated flour with different doses of pirimiphos-methyl (i.e., 0.1, 0.2, and 0.4 ppm) until pupation. The percentage of malformed larvae of *T. castaneum* was higher than *T. confusum*, while the reverse was observed in abnormal adults that exhibited incomplete elytra at 0.4 ppm (i.e., 22% vs. 24% for *T. castaneum* and *T. confusum*, respectively). However, Kamaruzzaman et al. (2006) demonstrated the direct effects of pirimiphos-methyl on the larval development of both species when reared on treated food. Based on the results of the current study, exposure intervals > 36 h of parental *T. confusum* females to the treated concrete are needed to affect the morphology of progeny.

The current study indicates that the effects of α-cypermethrin and pirimiphos-methyl on the elytra shape changes of *T. castaneum* and *T. confusum* progeny are not significant and this was determined for both sexes. In contrast, these insecticides have an almost equally strong effect on the hindwing deformities of *T. castaneum* and slightly more in male than in female offspring. The hindwings of *T. confusum* did not respond to both insecticide treatments. Although *T. castaneum* and *T. confusum* are biologically and morphologically closely related stored-product pests, they do not suffer similar indirect effects when treated with the same insecticides. Therefore, from a practical point of view, the fact that *T. confusum* appears more tolerant than *T. castaneum* under the tested scenarios should differentiate the management strategies between the two species. Morphological anomalies appearing in offspring, whose parents experienced treatments with insecticides, could moderate the frequency of insecticidal applications in storage facilities.

## Data Availability

The datasets used and/or analyzed during the current study are available from the corresponding author on reasonable request.
